# Production of Bioactive Compounds by Food Associated *Galactomyces geotrichum* 38, as Determined by Proteome Analysis

**DOI:** 10.3390/nu11020471

**Published:** 2019-02-23

**Authors:** Anna Grygier, Kamila Myszka, Artur Szwengiel, Kinga Stuper-Szablewska, Joanna Pawlicka-Kaczorowska, Grażyna Chwatko, Magdalena Rudzińska

**Affiliations:** 1 Institute of Food Technology of Plant Origin, University of Life Sciences, Wojska Polskiego 31, 60-624 Poznań, Poland; artursz@up.poznan.pl (A.S.); magdar@up.poznan.pl (M.R.); 2 Department of Biotechnology and Food Microbiology, University of Life Sciences, Wojska Polskiego 48, 60-627 Poznań, Poland; kmyszka@up.poznan.pl (K.M.); joannap@up.poznan.pl (J.P.-K.); 3 Department of Chemistry, University of Life Sciences, Wojska Polskiego 75, 60-625 Poznań, Poland; kstuper@up.poznan.pl; 4 Department of Environmental Chemistry, University of Łódź, Pomorska 163, 90-236 Łódź, Poland; grazyna.chwatko@chemia.uni.lodz.pl

**Keywords:** *G. geotrichum*, ergosterol, vitamin B_2_, trehalose, protein analysis

## Abstract

Fried cottage cheese is a dairy product, popular in some parts of Poland. Proteomic analysis of a culture of the mold *Galactomyces geotrichum* 38 isolated from fried cottage cheese was performed using UHPLC/MS. From the proteins identified, we selected those involved in the biosynthesis of bioactive compounds and those useful in industry. In the *G. geotrichum* 38 culture, the production quantities of vitamin B_2_ (224 μg/L), ergosterol (54.63 mg/kg), and trehalose (0.91 g/L) were determined by HPLC. The identified proteins were also used to prepare a hypothetical fatty acid biosynthesis pathway, and the percentage of individual sphingolipids in the culture was determined. Sphingolipids are also bioactive compounds. During culturing of *G. geotrichum* 38, the percentage of three sphingolipids increased. The last step of the research was to prepare a model of fried cottage cheese. The mold *G. geotrichum* 38, used in the process of ripening fried cottage cheese, synthesized vitamin B_2_ and erogsterol, which influenced the nutritional value of the product.

## 1. Introduction

Many microorganisms are capable of producing bioactive compounds [1], and only some of these possibilities are currently known. The mold *Galactomyces geotrichum*, or *G. geotrichum,* is a little-known microorganism that is used as a starter or nonstarter culture in the production of many cheeses throughout the world [2]. The literature indicates that *G. geotrichum* is capable of producing peptides that inhibit angiotensin I converting enzyme [3] and polyunsaturated fatty acids (PUFA) [4]. 

Proteomics is an approach that can help in understanding the ability of a microorganism to produce bioactive compounds. Proteomics involves the analysis of the composition, structure, and function, of proteins and of the interactions that take place between them. Such research can be carried out to identify microbial strains, to analyze proteins, and to find factors determining pathogenicity and interactions with the host. Such information about proteins can lead to knowledge of the microorganism’s ability to produce bioactive compounds [5]. There are no reports in the literature describing the proteome of *G. geotrichum*, but on the basis of the proteomics carried out on *G. geotrichum* 38, the mold’s ability to biosynthesize vitamin B2, ergosterol, sphingolipids, and lipoic acid was analyzed. Vitamin B_2_ is an important component that affects the proper functioning of the eye and has a role in amino acid and fat metabolism [6,7]. Another bioactive compound that can be produced by microorganisms is ergosterol, the main precursor to cortisone and the hormone progesterone [8]. Sterols are important in bone metabolic processes and in the regulation of calcium homeostasis [9]. Balanced sterols in the diet ensure optimal functioning of many organs and systems, including the kidneys, skin, skeletal muscles, cardiovascular system, immune system, nervous system, and endocrine systems [10]. Publications on ergosterol are most often associated with the study of the contamination of grain by fungi. 

Other bioactive compounds include sphingolipids, which are structural elements of the biological membranes of eukaryotic microbes [11]. They also participate in the control of autophagy, which occurs during thermal stress [12]. Sphingolipids inhibit colon carcinogenesis and regulate cholesterol levels [13]. Lipoic acid is a cofactor widespread in the majority of prokaryotic and eukaryotic microorganisms, as well as in plant and animal tissues [14]. It is endogenously synthesized in the liver, though not in sufficient quantities to meet the body’s needs. Lipoic acid has a role in the treatment of diseases in which free radicals are important for membrane phospholipids (e.g., liver diseases, neurological disorders, and diabetes). It is a universal antioxidant under conditions of oxidative stress [14]. Some microorganisms also produce trehalose, a saccharide made of two molecules of glucose. This compound has no reducing properties, which allows it to be used in food technology for sweetening products that will be subjected to heat treatment, as it does not cause browning reactions [15]. In molds, trehalose is a component of the cell membrane and occurs as a spare saccharide, while also stabilizing proteins [16]. 

*G. geotrichum* 38 is capable of producing bioactive compounds. In our study, proteomic analysis identified the enzymes used by *G. geotrichum* 38 to biosynthesize bioactive compounds. The potential of *G. geotrichum* 38 to biosynthesize sphingolipids, vitamin B2, ergosterol, and trehalose has not yet been studied. The production of bioactive compounds by *G. geotrichum* 38 can be used to prepare fried cheese enriched with bioactive compounds due to the presence of *G. geotrichum* 38.

## 2. Material and Methods

### 2.1. Reagents

Potassium phosphate, potassium chloride, glycerol, bicinchoninic acid, Triton X-100, sodium deoxycholate, trypsin, chloroform, methanol, ammonium formate, formic acid, acetonitryle, pentane, tris-(2-carboxyethyl) phosphine, hydrochloride (TCEP), acetic acid, vitamin B_12_, sulfuric acid (II), ergosterol, lipoic acid and vitamin B_2_ standard were procured from Sigma Aldrich (Saint Louis, MO, USA). A solution of 1M potassium hydroxide in methanol was purchased from Fluka (Saint Louis, MO, USA). Chloric acid (VII) was obtained from J.T. Baker (Denventer, Netherlands). Glucose, MgSO_4,_ ZnSO_4,_ FeSO_4_, HCl and NaOH were purchased from Avantor (Gliwice, Poland). Yeast extract was obtained from BD (NJ, USA). K_2_HPO_4_ was purchased from PPH Standard (Lublin, Poland). MnSO_4_ was procured from Chempur (Piekary Śląskie, Poland). 1-benzyl-2-chloropyridinium bromide (BCBP) was synthesized in the Department of Environmental Chemistry, University of Łódź, in accordance with the procedure described in Bald et al. [17]. Rapeseed oil (ZT Kruszwica S.A., Kruszwica, Poland) was purchased at a local market.

### 2.2. Microorganisms and Cultures

The organism used in this study, *G. geotrichum* 38, has been described by Grygier et al. [3]. For the first proteomic analysis, *G. geotrichum* 38 was cultured on a medium in 250 mL flasks (glucose 2%, yeast extract 1%) [18]. The culture was grown under dynamic conditions (100 rpm) at 30 °C for 96 h. For the second proteomic analysis, the culture was grown in a Biostat A plus bioreactor (Sartorius, Gottingen, Germany) with a capacity of 5 L. The cultivation was carried out under dynamic conditions (100 rpm) at 30 °C for 214 h. The cultures were aerated at an intensity of 1.5 vvm (gas volume flow per unit of liquid volume per minute). The pH value of the environment was 6.5. The medium consisted of 10 g/L rapeseed oil, 5 g/L yeast extract, 0.05 g/L K_2_HPO_4_, 0.17 g/L MgSO_4_, 0.015 g/L MnSO_4_, 0.015 g/L ZnSO_4_, 0.05 g/L FeSO_4_, and 10 mg/L vitamin B12. The above culture conditions stimulated the biosynthesis of PUFA by *G. geotrichum* 38. To prevent contamination of the cultures, 100 µL cultures were placed on Petri dishes and incubated at 30 °C once every two days. The Petri dishes were monitored for the presence of contamination.

### 2.3. Identification of Proteins Produced by G. geotrichum 38

The supernatant obtained from the *G. geotrichum* 38 culture was filtered through a 0.45 μm filter (GVS Filter Technology, Roma, Italy). The samples were concentrated five times on an Amicon filter (Merck Millipore, Burlington, MA, USA), washing the filter ten times with a buffer consisting of 25 mM potassium phosphate solution, 100 mM potassium chloride solution at pH 7.0, and 10% glycerol solution. The condensed supernatant was analyzed by sodium dodecyl sulfate polyacrylamide gel electrophoresis (SDS-PAGE). The protein concentration in the assay was determined by the spectrophotometric method using bicinchoninic acid (BCA) reagent [19]. To extract the intracellular proteins, the *G. geotrichum* sediment was suspended in a buffer composed of: 25 mM potassium phosphate, 100 mM potassium chloride solution at pH 7.0, and a 10% glycerol solution. The samples were then mixed on a glass vortex shaker (Sigma Aldrich, Saint Louis, MI, USA). A 1% solution of Triton X-100 and a 1% solution of sodium deoxycholate were introduced into the tests. Incubation of the samples was carried out at 4 °C for 1 h. The samples were then centrifuged to separate the soluble and insoluble fraction of proteins. Soluble proteins from *G. geotrichum* 38 cells were analyzed by SDS-PAGE and spectrophotometric reagents with BCA reagent. The extracellular and intracellular proteins of *G. geotrichum* 38 were then subjected to proteolysis using trypsin [20]. Specific peptides obtained from proteolysis were analyzed using UHPLC/MS (Thermo Fischer Scientific, Waltham, MA, USA) and the results compared with the MASCOT database (minimum significant score of <68, significance threshold *p* < 0.05, ions score or expect cut-off: 30). MS data processing was based on Celińska et al. [20]. The analyses were carried out in the laboratory of the Blirt company (Gdańsk, Poland).

### 2.4. Determination of Sphingolipid Content

The method of Singh and Del Poeta [12] was used to determine the content of sphingolipids in the *G. geotrichum* 38 cell biomass. The samples were frozen at -20 °C until needed for analysis. Determinations were carried out in triplicate. Sphingolipid content was determined by reversed phase chromatography using mass spectroscopy. The analysis was carried out on a Dionex UltiMate 3000 Ultra-Performance Liquid Chromatography (UHPLC) (Thermo Fisher Scientific, Waltham, MA, USA) device coupled with an ultrahigh-resolution Bruker maXis (Bruker, Billerica, MA, USA) tandem spectrometer using a quadrupole time-of-flight analyzer. Synergi 4 μm Fusion-RP 80 Å column, LC column, 150 × 3.0 mm (Phenomenex, Torrance, CA, USA) were used. Water as the mobile phase contained 2 mM HCOONH_4_ and 0.2% formic acid (component A). Component B was methanol with 2 mM HCOONH_4_ and 0.2% formic acid. The injection volume was 10 μL and the flow rate was 0.3 mL/min with an elution gradient of 50% to 80% of component B in 10 min, further to 99% of component B in 20 min, and holding under these conditions for 20 min. The chromatography column was thermostated at 40 °C. To identify compounds, MS spectra were recorded using electrospray ionization (ESI) as the positive ion mode with the electrospray ionization method (target analysis). The mass spectrometer operation parameters with ESI source were capillary voltage at 4500 V, nitrogen nebulization at 1.8 bar pressure, and drying gas flow of N_2_ at 9 L/min at 200 °C. The ionic signal was collected in the 80–1200 *m*/*z* range. Gradient elution was determined according to the method of Zhang et al. [21]. The ESI-MS system was calibrated using sodium formate salt. The molecular weight standard was introduced at the beginning of chromatographic separation. Data Analysis 4.1 (Bruker Daltonik, Hamburg, Germany) and Profile Analysis (Bruker Daltonik) software were used to analyze the results. The chromatogram extracts peaks corresponded to ions of the analyzed compounds [M + H]^+^ [12]. The compounds found in the samples were identified based on the molecular weight of the parent ion (MS experiment) and on structural information from mass spectrometer (MS/MS experiment).

### 2.5. Determination of B Vitamin Content

The amount of B vitamins synthesized by *G. geotrichum* 38 in the culture was evaluated by reversed phase chromatography and mass spectroscopy using a Dionex UltiMate 3000 UHPLC device coupled with a Bruker maXis tandem spectrometer. The modified methodology proposed by Zand et al. [22] and a standard of vitamin B_2_ were used. A Kinetex 1.7 μm C18 100 Å LC column 100 × 2.1 mm was used. The mobile phases were 0.1% formic acid in water (A) and 0.1% formic acid in acetonitrile (B). Gradient conditions used were: for the first five min, the share of component B was 5%, increasing linearly over 9 min to 90%. The flow rate was 0.2 mL/min. The volume of the injected sample was 20 μL. The column was thermostated at 40 °C. Ionization was carried out using the ESI method in positive ion mode, [M + H]^+^ (target analysis). The results obtained were read from the Data Analysis 4.1 program. During the MS/MS experiments, compounds were detected using molecular weight and structural information from the detector with the help of the Kyoto Encyclopedia of Genes and Genomes (https://www.genome.jp/kegg/). The analysis was carried out in triplicate.

### 2.6. Determination of Sterol Content of G. geotrichum 38 Biomass

The sterols content of the *G. geotrichum* 38 biomass was determined according to the method of Perkowski et al. [23]. From the sample, 0.1 g biomass was taken and 2 mL of methanol and 0.5 mL 2 M NaOH were added. The test tubes were placed in closed plastic bottles, which were placed inside a microwave oven. The samples were irradiated for 20 s at a power of 370 W. After 5 min, the process was repeated. After cooling, the samples were neutralized with 1 mL 1 M aqueous HCl and 2 mL methanol. Extraction was carried out using pentane (3 × 4 mL). Methanol was added once the pentane had evaporated. The samples were then treated with ultrasound. The sterols were analyzed using HPLC with a UV detector at a wavelength of 282 nm. A Nova Pak C-18 column (4 μm, 150 × 3.9 mm) (Waters, Milford, MA, USA) was used for separation [23]. An ergosterol standard was used. Elution was carried out in a methanol:acetonitrile (90:10) system at a flow rate of 0.6 mL/min. The volume of the injected sample was 50 μL. The determinations were carried out in triplicate.

### 2.7. Determination of Lipoic Acid Content

The lipoic acid contents were determined following Chwatko et al. [24]. Each sample was prepared in five replications. A liquid chromatograph from Hewlett–Packard (Palo Alto, CA, USA) was used and separation was carried out on a Zorbax SB-C18 column (150 mm × 4.6 mm, 5 μm) from Agilent Technologies (Santa Clara, CA, USA), using a gradient elution with the following profile: 0–5 min: 10%–40% B; 5–6 min: 40%–10% B; 6–8 min: 10% B. Component A of the mobile phase was 2% acetic acid, while component B was acetonitrile. The column temperature was 25 °C, the mobile phase flow rate was 1 mL/min through the column, and the analytical wavelength was 321 nm. The volume of the injected sample was 5 μL. Lipoic acid was used as a standard.

### 2.8. Determination of Trehalose Content of Culture

The trehalose content of the *G. geotrichum* 38 culture was determined following the method described by Pawlicka et al. [25]. The cultures were centrifuged at 4500 g/10 min. The supernatant was then decanted, and the cellular biomass was washed with distilled water and again centrifuged (4500 g/10 min). The pellet was resuspended in 1.6 mL of an 80% methanol solution (v/v). The samples were next incubated for an hour at 60 °C. The samples were analyzed using a HPLC Agilent Technologies 1200. The analysis used a 300 × 7.8 mm Rezex ROA column (Phenomenex, Torrance, CA, USA), which was thermostated at 40 °C. A 0.001 M H_2_SO_4_ solution with a flow rate of 0.6 mL/min was used as the mobile phase. The volume of the injected sample was 50 μL. A trehalose standard was used to identify the samples and as an external standard to quantify analysis. The samples were analyzed in triplicate.

### 2.9. Production of Fried Cottage Cheese Using G. geotrichum 38 and Determination of Vitamin B_2_ and Ergosterol

Fried cottage cheese was produced by combining cottage cheese with *G. geotrichum* 38 biomass and leaving the mixture at room temperature for 3 days, before being pan-fried with butter. The production of fried cottage cheese using *G. geotrichum* 38 has been described by Grygier et al. [4].

The quantity of vitamin B_2_ in milk was determined following Schmidt et al. [26]. 1M HCl solution was added dropwise to 10 mL of milk until a pH of 4.0 was obtained. Water was then added to a volume of 30 mL. The samples were incubated for 10 min at 20 °C with constant shaking. The samples were further centrifuged (4000 g/10 min) and the resulting supernatant was filtered through a syringe filter (0.2 μm). This was measured for the cheese using the methodology described by Stancher and Zonta [27]. To 5 g of finely chopped fried cheese, obtained after 72 h of ripening, 7 mL of methanol:water (1:2 *v/v*) solution was added. The samples were shaken for 4 min, and 3 mL of glacial acetic acid was added before shaking again. After the samples were centrifuged (2000 g/15 min), the supernatant was transferred to a 20 mL graduated flask. The remaining precipitate was washed three times with 4 mL of water:methanol:glacial acetic acid (65:25:10 *v/v/v*). The solution was then added to 20 mL of the sample. The samples were filtered through a syringe filter (0.2 μL). Trials of milk and cheese were performed in triplicate. Chromatographic analysis of vitamin B_2_ was performed as in Section 2.5. The ergosterol content of cheese was determined in accordance with the methodology described in the Section 2.6.

## 3. Results and Discussion

### 3.1. Proteomic Analysis of Proteins Synthesized by G. geotrichum 38

A total of 27 proteins were extracted and identified from hyphae of *G. geotrichum* 38 cultured on a medium as described by Bajpai et al. [18]. Table 1 lists the identified molecules. Most of these proteins are molecules involved in the cell division cycle associated with DNA replication. The present study also identified the α-subunit of the acetyl-CoA carboxyl transferase protein (ACC); this is an enzyme that catalyzes the carboxylation of acetyl-CoA to malonyl-CoA [28] as the first stage in fatty acid biosynthesis. The resulting malonyl-CoA is used in the synthesis of long-chain fatty acids [28]. In yeast, the deletion of the ACC enzyme gene is lethal. Even the presence of fatty acids in the medium does not allow cell survival in the absence of the ACC enzyme. This confirms the crucial role of the ACC enzyme in the biosynthesis of fatty acids. The synthesized fatty acids may act as components of sphingolipids and ceramides [28]. In the study of Besada-Lombana et al. [29] on the yeast *S. cerevisiae*, overexpression of the ACC gene guaranteed an increase in the efficiency of fatty acid synthesis, in particular of oleic acid. Also, an increase of lipid content of *E. coli* cells was found upon overexpression of the ACC enzyme gene [30]. The presence of the ACC enzyme in the fungal cells of *G. geotrichum* 38 confirms the ability of this microorganism to biosynthesize fatty acids. Grygier et al. [4] demonstrated the ability of *G. geotrichum* 38 to produce PUFA in culture medium and in fried cottage cheese. The presence of PUFA in the body can help prevent many diseases, particularly those of the circulatory system. The body does not produce PUFA, so they should be taken with food [31]. The confirmation of the production of PUFA by *G. geotrichum* 38 points the way to preparing dairy products with increased levels of PUFA.

Protein expression depends on the growth conditions of microorganisms; the proteins of *G. geotrichum* 38 were, therefore, characterized again on a medium that allowed an increase in the production of polyunsaturated fatty acids. A total of 218 proteins were found in cellular biomass. A total of 206 proteins were found in the supernatant. Forty proteins were found in both the cellular biomass and the supernatant. The proteins synthesized by *G. geotrichum* 38 consist of proteins related to the cell life cycle and proteins involved in biochemical transformations, on the basis of which bioactive compounds are synthesized. A third group of proteins are molecules whose biological/biotechnological function is unknown. Some proteins and their functions are presented in Table 2. Proteomic analysis made it possible to identify the enzymes necessary for the biosynthesis of bioactive compounds [51]. This study highlighted the ability of *G. geotrichum* 38 to biosynthesize five bioactive compounds. There have not yet been any reports in the literature on the production of sphingolipids, B-vitamins, trehalose, ergosterol, nor lipoic acid by *G. geotrichum*. The introduction to this study describes the importance of these compounds for health and for industry.

### 3.2. Hypothetical Fatty Acid Biosynthesis Pathway

**Figure 1:**
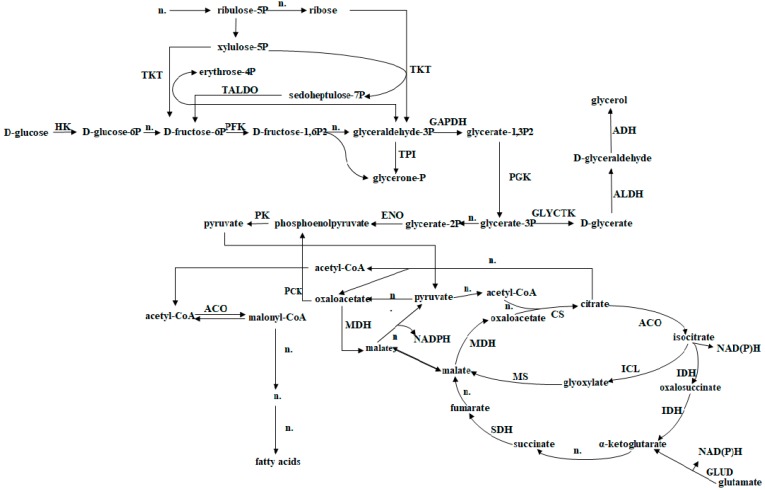
Pathway of fatty acid transformation in *G. geotrichum*. hexokinase (HK, EC2.7.1.1), transketolase (TKT, EC2.2.1.1), transaldolase (TALDO, EC2.2.1.2), phosphofructokinase (PFK, EC2.7.1.11), glyceraldehyde 3-phosphate dehydrogenase (GAPDH, EC1.2.1.12), phosphoglycerate kinase (PGK, EC2.7.2.3), triose-phosphate isomerase (TPI, EC5.3.1.1), enolase (ENO, EC4.2.1.11), pyruvate kinase (PK, EC2.7.1.40), malate dehydrogenase (MDH, EC1.1.1.37), citrate synthase (CS, EC2.3.3.1), aconitase (ACO, EC4.2.1.3), isocitrate dehydrogenase (IDH. EC1.1.1.41 and EC1.1.1.42), glutamate dehydrogenase (GLUD, EC1.4.1.2 and EC1.4.1.3), phosphoenolpyruvate carboxykinase (PCK, and EC4.1.1.32 EC4.1.1.49), succinate dehydrogenase (SDH EC1.3.5.1), alcohol dehydrogenase (ADH, EC1.1.1.2), phosphoglycerate kinase (GLYCTK, EC2.7.1.31), aldehyde dehydrogenase (ALDH, EC1.2.1.3), isocitrate lyase (ICL, EC4.1.3.1), methionine synthase (MS, EC2.1.1.13), and n.- enzymes, whose content in the sample cannot be identified the method used (own work on the basis of Wang et al. [63]).

Certain enzymes found in the proteome of *G. geotrichum* 38 may be associated with the biosynthesis of fatty acids—namely, citrate ATP lyase and isocitrate dehydrogenase. Acetyl-CoA carboxyl transferase enzyme and aconitase were also found. The biosynthesis of these enzymes is crucial for the production of fatty acids. Aconitase catalyze the conversion of acetyl-CoA to malonyl-CoA, which begins the fatty acid biosynthesis pathway [63]. In *G. geotrichum* 38, this pathway begins with gluconeogenesis, which requires a carbon source to be available in the nutrient medium. This process involves the enzymatic conversion of nonsaccharide precursors into glucose [63]. Glycerol, derived from rapeseed oil, may act as such a precursor. In glyoxysomes, lipids are transformed into saccharides. This confirms the presence of isocitrate lyase and malate synthase enzymes. The end product of gluconeogenesis is pyruvate, which participates further in the Krebs cycle. The citric acid formed at this stage is converted by ATP citrate lyase to acetyl-CoA, which is used in the synthesis of fatty acids [63]. On the basis of the proteome analysis of the biomass and the supernatant of *G. geotrichum* 38 culture on a substrate that stimulates PUFA biosynthesis, we can conclude that *G. geotrichum* 38 possesses a fatty acid biosynthesis pathway. The fatty acid biosynthesis pathway was presented in Figure 1. To date, the proteome of mold has only been described by Wang et al. [64]. Based on proteomic analysis, these researchers characterized the lipid transformation pathway in the mold *Mortierella alpina*, which has demonstrated the ability to produce fatty acids, triacylglycerols (TAGs), sterols, sphingolipids, and glycerophospholipids. Hamid et al. [65] demonstrated the effect of the enzymes 6-phosphogluconate dehydrogenase, glucose-6-phosphogluconate dehydrogenase, isocitrate dehydrogenase, malic enzyme, citrate ATP lyase, and fatty acid synthase on lipid accumulation in *Cunninghamella* fungal cells. These enzymes were synthesized under conditions of limited availability of nitrogen compounds. According to Hamid et al. [65], lipogenesis was possible in the presence of all six enzymes, with the activity of citrate ATP citrate, malic enzyme, and fatty acid synthase being particularly important [65].

### 3.3. Sphingolipids in G. geotrichum 38 Culture

Delta-8-fatty-acid desaturase was found in the proteome of *G. geotrichum* 38; this is responsible for the introduction of a double bond in long-chain sphingolipids and in fatty acids [52]. The *de novo* biosynthesis of sphingolipids begins with the condensation of L-serine and palmitoyl-CoA. This allows the production of 3-ketodihydrosfingosine, which is then reduced to dihydrosfingosine. Then, Δ-8 desaturase converts the dihydrosfingosine to Δ4-ceramide; Δ4, Δ8-ceramide (skeleton d18:2, 4,8-sphingodienyes) [11]. We searched the *G. geotrichum* 38 samples for the sphingolipids identified in other fungi species cultures [12]. Based on ion molecular weight, seven sphingolipids were found: 4-sphingenine (d18:1), phytosphingosine (t18:0), 4,8-sphingadiene (d18:2), 9-methyl-4,8-sphingadiene (d19:2), 4-amino-9-methyl-4,8-nonadecadiene-1,3-diol (d20:2), 4-hydroxy-9-methyl-1-4,8-sphingadiene (d19:2_OH_), 4-amino-9-methyl-8-nonadecene-1,3,4-triol (d20:1). The percentage occurrence of each is shown in Table 3. The profile was dominated by 4-amino-9-methyl-8-nonadecene-1,3,4-triol (d20:1). The levels sphingolipids were measured twice in order to determine any quantitative changes that occurred during culturing. During culturing of *G. geotrichum* 38, the percentage of 4-sphingenine, phytosphingosine, and 4-amino-9-methyl-8-nonadecene-1,3,4-triol increased. In those samples, the levels of 4,8-sphingadiene, 9-methyl-4,8-sphingadiene, 4-amino-9-methyl-4,8-nonadecadiene-1,3-diol, and 4-hydroxy-9-methyl-1-4,8-sphingadiene decreased in the sphingolipid profile with increasing culture time. This may have been due to the use of glycerol from these compounds for the esterification of *de novo* fatty acids [66]. As seems to be the case from the available literature, microorganisms synthesize mainly 9-methyl-4,8-sphingadiene. The main producers of this compound are *Cryptococcus* spp., *Aspergillus* spp., and *Candida* spp. [12]. 

### 3.4. Determination of B-Group Vitamins in G. geotrichum 38 Culture

A pyridoxine biosynthesis PDX1-like enzyme was found in the proteome of *G. geotrichum* 38, which is responsible for the biosynthesis of vitamin B_6_. Analysis confirmed the presence of vitamin B_6_, though this was derived from yeast extract—no increase occurred in the amount of vitamin B_6_ during cultivation. We did, however, find an increase in vitamin B_2_, whose precursor is guanosine-5′-triphosphate (GTP). The GTPase activating enzymes were present in the proteome of *G. geotrichum* 38. In mold, whose carbon source is fatty acids, biosynthesis begins in the peroxisomes. The glyoxylate cycle transforms it into GTP. GTP with serine are involved in three reactions that lead to the synthesis of 6,7-dimethyl-8-ribityllumazine. This compound is a precursor to vitamin B_2_ in the last stage of biosynthesis [67].

Table 4 presents the results of the yield determination of vitamin B_2_ biosynthesis by *G. geotrichum* 38. In the medium, at the start of *G. geotrichum* 38 culture, there was an average of 92 μg/L vitamin B_2_, derived from yeast extract. This value increased during the culture. After 216 h in the culture, 224 μg/L of vitamin B2 was obtained.

Similar trends were also observed by Stahmann et al. [68]. Mold of the species *Ashbya gossypii* was grown on nutrients supplemented with vegetable oil. This favored the biosynthesis of vitamin B_2_ by *Ashbya* spp. [68]. Optimal conditions for the overproduction of vitamin B_2_ by *Ashbya gossypii* included a temperature of 26–28 °C and oxygenation of the culture. Initially, the average yield of vitamin B_2_ by *Ashbya gossypii* was 200 mg/L. 

### 3.5. Determination of Sterols in G. geotrichum 38 Biomass

Analysis of the *G. geotrichum* 38 proteome revealed the presence of the protein YMR134W, which participates in ergosterol biosynthesis. In mold, the biosynthesis of ergosterol begins with the transformation of acetyl-CoA into isopentyl pyrophosphate. After the condensation reaction and the addition of further molecules of isopentyl pyrophosphate, squalene (a sterol precursor) is produced. Enzymes of the cytochrome P450 group are required in the biosynthesis of ergosterol [9]. Ergosterol was also found in the hyphae of *G. geotrichum* 38. In 96 h of culture, its yield within the cells was on average 54.63 mg/kg of dry biomass. Contreras et al. [69] examined the yeast *Xanthophyllomyces dendrorhous* for ergosterol production. The maximum ergosterol yield in the strains they tested was 4.21 mg/g of dry biomass [69]. Effective ergosterol biosynthesis is also possible using *Saccharomyces cerevisiae* and *S. uvarum* [8,70]. Nahlik et al. [8] found that the amount of ergosterol obtained depends on the final amount of cellular biomass. In this study, ergosterol production also increased with oxidative stress. The studied microorganisms were cultured on a medium supplemented with glucose and ethanol, which resulted in three times higher ergosterol values in culture (103.84 × 10^−6^ g/L h) than in the control sample [8]. 

### 3.6. Determination of Lipoic Acid Content of G. geotrichum 38 Culture

Levels of lipoic acid in microorganisms are correlated with their metabolic activity. Higher levels of lipoic acid are found when a pyruvate dehydrogenase complex is present in the cell. The main function of lipoic acid is thus to oxidatively decarboxylate pyruvate. In some microorganisms, it can degrade branched chain amino acids. A number of saprophytic food microorganisms exhibit the potential for lipoic acid biosynthesis; these are mainly representatives of the genera *Bacillus, Pseudomonas, Pedicoccus, Rhodospirillum, Anacystis, Gloecaps, Nostoc,* and *Saccharomyces* [71]. Mitochondrial lipoyl synthase enzyme was found, in the proteome of *G. geotrichum* 38; this is responsible for the synthesis of lipoic acid. However, no lipoic acid was found in the culture of *G. geotrichum* 38. It is possible that lipoic acid does not occur freely in cells, but is connected by an amide bond to the amino group of lysine found in proteins [72].

### 3.7. Determination of the Trehalose Content of G. geotrichum 38 Culture

**Figure 2:**
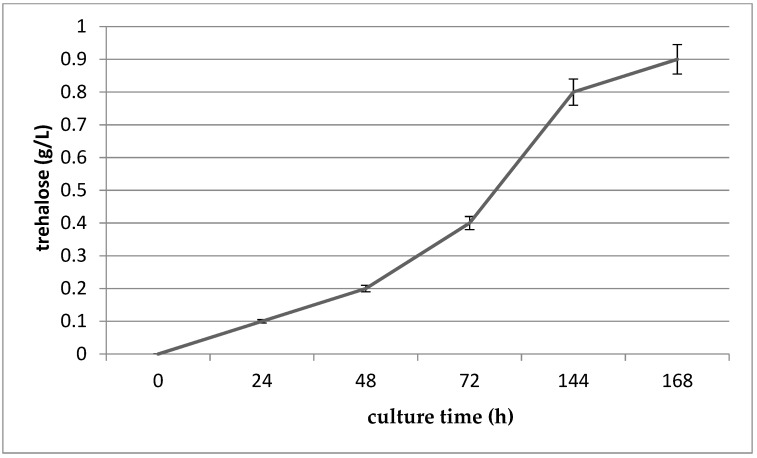
Trehalose content of *G. geotrichum* 38 culture.

The enzyme alpha,alpha-trehalose-phosphate synthase was found in the proteome of *G. geotrichum* 38. This enzyme catalyzes the transfer of glucose from uridine diphosphate (UDPglucose) to glucose-6-phosphate to form trehalose-6-phosphate. This step is followed by the hydrolysis of trehalose-6-phosphate to trehalose. The enzyme trehalose-6-phosphate phosphatase is involved in the above process [15]. The trehalose content of the culture systematically increased, and after 168 h of culture had reached 0.91 g/L (Figure 2). One microorganism known to be capable of biosynthesizing trehalose is *Propionibacterium* spp. The agents that stimulates trehalose biosynthesis by *Propionibacterium* spp. are lactose [73] and glycerol [74].

### 3.8. Vitamin B_2_ and Ergosterol in Fried Cottage Cheese Produced by G. geotrichum 38

The above experiments suggested determining vitamin B_2_ and ergosterol in a model food product, in a technological process used for the studied microorganisms. These compounds were determined in fried cottage cheese prepared after five days of digestion. Raw milk may also be a source of vitamin B_2_ in food products [27]. The levels of this compound were also evaluated in the raw material used in the technological process. In the experiments, the vitamin B_2_ content of the raw milk was 1.19 μg/g milk. The amount of the compound in fried cheese doubled, reaching an average of 3.50 μg/g cheese. The increase in this compound in the fried cottage cheese was most likely associated with the biosynthesis of vitamin B_2_ by *G. geotrichum* 38. In studies carried out on cheese produced in Italy, the vitamin B_2_ content ranged from 1.17 to 3.75 g/g of cheese [28]. The presence of ergosterol was also evaluated in fried cottage cheese, confirming the presence of ergosterol in the model food product. The amount of this compound in the final product was 0.78 mg/kg of cheese on average. To date, the presence of ergosterol in food products has been associated with food contamination with pathogenic microflora. The literature on the subject, however, lacks information describing the effect of starter and nonstarter microorganisms on increasing the ergosterol content of food. The fatty acid content has been presented in Grygier et al. [4]. No lipoic acid was determined in the fried cheese due to it being absent from the culture. 

## 4. Conclusions

In this study, we carried out proteomic analysis of a *G. geotrichum* 38 culture and selected those proteins that can participate in the biosynthesis of bioactive compounds (delta(8)-fatty-acid desaturase, pyridoxine biosynthesis PDX1-like protein, uncharacterized protein YMR134W, alpha,alpha-trehalose-phosphate synthase, lipoyl synthase, mitochondrial). *G. geotrichum* produced lipids containing long-chain polyunsaturated fatty acids. Additionally, we demonstrated the biosynthesis of vitamin B_2_, ergosterol, sphingolipids, and trehalose by *G. geotrichum* 38. When *G. geotrichum* 38 was used to prepare model fried cottage cheese, increases were seen in vitamin B_2_ and ergosterol levels. These results increase our knowledge of the potential of *G. geotrichum* to produce bioactive compounds and demonstrate the possibility of using it in the production of food products.

## Figures and Tables

**Table 1:** Proteins isolated from hyphae and culture of *G. geotrichum* 38.

**Proteins Extracted from Hyphae**	**Possible Function**
extracellular ligand-binding receptor	extracellular signal molecule [32]
radical sterile alpha motif domain protein	reducing S-adenosyl L-methionine [33]
peptidyl-tRNA hydrolase	cell cycle [34]
flavin adenine dinucleotide dependent oxidoreductase	catalyzing the oxidation of amino acids [35]
ATP-dependent DNA helicase Rep	cell cycle [36]
glutaredoxin C3	electron carrier in the synthesis of deoxyribonucleotides [37]
protein kinase C inhibitor 1	inhibiting protein kinase C activity [38]
excinuclease ATP-binding cassette subunit C	repairing DNA [39]
nicotinate (nicotinamide) nucleotide adenylyltransferase	metabolism of nicotine and nicotinamide [40]
acetyl co-enzyme A carboxylase carboxyltransferase alpha subunit	biosynthesis of fatty acids [28]
Silent Information Regulator A family protein	inducing arthrospore creation [41]
pyruvate carboxyltransferase	carboxylation of pyruvate [42]
methionine sulfoxide reductase A	reducing methionine sulfoxide to methionine [43]
transposase for IS660	cell cycle [44]
transposase, IS4 family	cell cycle [45]
**Proteins Extracted from Culture**	**Possible Function**
elongation factor thermo stable	cell cycle [46]
putative endolysin	catabolism of chitin [47]
cytochrome C biogenesis protein, cytochromes c maturation protein H family protein	hem lyase subunit [48]
helix-turn-helix, AraC domain protein	cell cycle [49]
heavy metal transport/detoxification protein	metal transport [50]
**Proteins with an Unidentified Function**	
hypothetical protein HMPREF7215_1957	
hypothetical protein bthur0004_61160	
hypothetical protein Veis_3482	
PREDICTED: similar to hCG1786587	
hypothetical protein XF2003	
hypothetical protein RPC_3615	
putative endolysin of prophage CP-933O	

**Table 2:** Proteins isolated from hyphae and culture of *G. geotrichum* 38 that are related to the biosynthesis of bioactive compounds and other metabolites of industrial importance.

**Proteins Extracted from Hyphae**	**Possible Protein Function**
delta(8)-fatty-acid desaturase	sphingolipid biosynthesis [52]
pyridoxine biosynthesis PDX1-like protein	biosynthesis of B-group vitamins [53]
saccharopine dehydrogenase	lysine biosynthesis [54]
enolase-phosphatase E1	methionine biosynthesis [55]
imidazole glycerol phosphate synthase hisHF	histidine biosynthesis [56]
**Proteins Extracted from Culture Fluid**	**Possible Protein Function**
pyridoxine biosynthesis PDX1-like protein	biosynthesis of B-group vitamins [53]
uncharacterized protein YMR134W	ergosterol biosynthesis [57]
alpha,alpha-trehalose-phosphate synthase	trehalose biosynthesis [58]
lipoyl synthase, mitochondrial	lipoic acid biosynthesis [59]
2-isopropylmalate synthase;3-isopropylmalate dehydrogenase	leucine biosynthesis [60]
amino-acid acetyltransferase, mitochondrial	arginine biosynthesis [61]
probable 5-methyltetrahydropteroyltriglutamatehomocysteine methyltransferase	methionine biosynthesis [62]

**Table 3:** Percentage of individual sphingolipids *G. geotrichum* 38 (**1.**) 4-sphingenine (d18:1), (**2.**) phytosphingosine (t18:0), (**3.**) 4,8-sphingadiene (d18:2), (**4.**) 9-methyl-4,8-sphingadiene (d19:2), (**5.**) 4-amino-9-methyl-4,8-nonadecadiene-1,3-diol (d20:2), (**6.**) 4-hydroxy-9-methyl 1-4,8-sphingadiene (d19:2_OH_), (**7.**) 4-amino-9-methyl-8-nonadecene-1,3,4-triol (d20:1)).

	Sphingolipid {%}						
Culture Time (h)	(1.)	(2.)	(3.)	(4.)	(5.)	(6.)	(7.)
48	25.7 ^a^ ± 1.0	0.2 ^a^ ± 0.0	1.1 ^a^ ± 0.0	0.5 ^b^ ± 0.0	8.7 ^b^ ± 0.1	3.0 ^b^ ± 0.0	60.8 ^a^ ± 1.7
216	28.2 ^b^ ± 0.3	0.9 ^b^ ± 0.0	0.6 ^a^ ± 0.1	0.2 ^a^ ± 0.0	5.9 ^a^ ± 0.1	0.3 ^a^ ± 0.0	63.9 ^b^ ± 0.5

Different letters within columns indicate significant differences at α = 0.05.

**Table 4:** Vitamin B_2_ content of *G. geotrichum* 38 culture.

Culture Time {h}	Vitamin B2 Content µg/L
0	92 ^a^ ± 4
216	224 ^b^ ± 10

Different letters within columns indicate significant differences at α = 0.05.
